# Inter-Individual Differences in Cognitive Tasks: Focusing on the Shaping of Decision-Making Strategies

**DOI:** 10.3389/fnbeh.2022.818746

**Published:** 2022-03-29

**Authors:** Elsa Pittaras, Héloïse Hamelin, Sylvie Granon

**Affiliations:** ^1^Heller Laboratory, Department of Biology, Stanford University, Stanford, CA, United States; ^2^Institut des Neurosciences Paris-Saclay, CNRS UMR 9197, Saclay, France

**Keywords:** gambling, monoamines, prefrontal cortex, mice models, individual variations

## Abstract

In this paper, we review recent (published and novel) data showing inter-individual variation in decision-making strategies established by mice in a gambling task (MGT for Mouse Gambling Task). It may look intriguing, at first, that congenic animals develop divergent behaviors. However, using large groups of mice, we show that individualities emerge in the MGT, with about 30% of healthy mice displaying risk-averse choices while about 20-25% of mice make risk-prone choices. These strategies are accompanied by different brain network mobilization and individual levels of regional -prefrontal and striatal- monoamines. We further illustrate three ecological ways that influence drastically cognitive strategies in healthy adult mice: sleep deprivation, sucrose or artificial sweetener exposure, and regular exposure to stimulating environments. Questioning how to unmask individual strategies, what are their neural/neurochemical bases and whether we can shape or reshape them with different environmental manipulations is of great value, first to understand how the brain may build flexible decisions, and second to study behavioral plasticity, in healthy adult, as well as in developing brains. The latter may open new avenues for the identification of vulnerability traits to adverse events, before the emergence of mental pathologies.

## Introduction

The reason why cognitive abilities differ between individuals is currently unknown. Cognitive performance depends on multiple processes such as motivation, emotions, learning, memory and social environment. These processes develop and evolve differently between individuals, as they rely on neural and neurochemical pathways that evolve, change, and mature with time due to the influence of genetic, environmental, social, and epigenetic mechanisms. Functional brain connectivity seems more important than brain activity itself, as revealed in a recent study showing that only 20 percent of variation between humans for intelligence scale can be explained by resting state brain activity while brain connectivity provided more information (Dubois et al., 2018). While brain activity provides an instant picture of brain function, it doesn’t provide information about stable brain function, nor about cellular or molecular mechanisms underlying activity. By contrast, brain connectivity has been shown to be extraordinarily plastic, and neural plasticity directly influences brain network organization (Stampanoni Bassi et al., 2019). Functional brain connectivity, which is very variable between individuals (Gallen and D’Esposito, 2019), has been shown to be altered by multiple life events, either long lasting such as practicing sports (Wang et al., 2016) or meditation (Sharp et al., 2018), or acute such as the use of a brain-computer-interface (Nierhaus et al., 2021).

Each individual establishes with the world a personal interaction: the way each of us focus on a specific cue, among the myriad of those around us, and internalizes it and its value, integrates it with other experiences, and involves it in our future decisions. Interindividual variation in decision making is of great interest because it can reveal differences in the brain’s way to solve the same problem. If one way or another stabilizes over time, it can become an overt trait of the individual or strategy. Studying interindividual differences in decision making might enable us to learn how pathological states may emerge in one individual but not in another, hence leading to personality traits such as vulnerability and resilience. Finally, studies of inter individual decision making can also allow us to study how personality may emerge, which environmental factors could help shaping them and whether individual (and collective) ability could be improved.

The inclusion of variation among individuals has been central to the study of human psychology since the early 20th century but that research on variability in animal models of brain function has been lacking until very recently. Many reasons can be offered for that fact (see Boogert et al., 2018 for a review). The first one may be that considering group effects is interesting from a statistical point of view, as results are likely to apply to a majority of individuals, providing that data follow a Gaussian type of distribution. The second reason relies on cost. Indeed, focusing on a limited number of individuals (i.e., those that fit with the mean of the group) reduces the number of subjects needed to get statistical significance. A third -more recent- reason may lie on the requested reduction of animals used in experimental research. However, despite the legitimate reasons that might exist, neglecting to address individual variations precludes first the understanding of the multiple possibilities for problem solving in healthy individuals, and secondly, prevents us from deciphering the parameters of vulnerability or resilience to adverse events amongst individuals. Hence, this neglect of consideration for inter-individual variation may well be one of the major causes for our inability to find effective treatments for brain pathologies. Another consequence of neglecting inter-individual variation is the elimination of consideration of the complexity of problem solving in animals. To obtain group data fast, animal research has mostly focused on fast behavioral tests that can be solved within minutes in a similar way by all individuals. Although this can be of interest in some cases (immediate reactivity, stress, attention), it prevents the study of how cognitive strategies emerge and evolve with time.

In this paper, we review and discuss inter-individual differences in decision making and the underlying neurobiological factors in mice. Our review reveals neural and neurochemical correlates of individual cognitive strategies. We then illustrate, with published and original data, ways to shape these strategies by manipulating environmental factors. Lastly, we explore whether this kind of approach can open new windows into the identification of vulnerability traits to pathological states.

## What Is Inter-Individual Variation?

### Behavioral Expression

Some choices are simple and bidirectional: there is an advantageous option and a disadvantageous one. In those situations, a large majority of healthy subjects choose the advantageous option, and the only difference between them may reside in time spent to make the choice. However, some choices are more ambiguous and complex: more uncertainty in the probability to get the reward, in the evaluation of its quality, and in the existence of a delay or in a cognitive effort to get it. Faced with such choices, inter-individual differences emerge, and subjects develop different strategies of decision-making (Bechara et al., 2002). Looking at the results as the mean of subjects’ performance may mask a lot of information (Parasuraman and Jiang, 2012). Indeed, studying inter-individual differences during cognitive tasks reveals different individual strategies, and may explain a portion of the variability observed on brain activity during fMRI studies (Kirchhoff and Buckner, 2006; Miller et al., 2012). Despite those studies, most scientific research maintains a whole group approach, neglecting inter-individual differences (Faigman, 2010). Moreover, studying inter-individual differences in cognitive performance may be a common goal for different fields, which need individual understanding of cognitive functions and behavior, such as the court of law (Faigman, 2010) and education (Posner and Rothbart, 2005), to make better decisions. This interest for inter-individual differences, which is beginning to be well developed in humans, may be extended to animal models. Indeed, some studies already show that inter-individual differences are observable in healthy or aged animal models such as non-human primates or rodents during cognitive tasks (Hok et al., 2012) or in pathological conditions. Those studies highlight that animals show different performances between individuals in learning or spatial memory (Bathellier et al., 2013; Maugard et al., 2019), decision-making (Pittaras et al., 2013, 2018; Pittaras E. et al., 2016), or show more vulnerability to develop pathology like neuropathic pain (Martinez-Navarro et al., 2019), alcohol abuse (Higley and Bennett, 1999), or post-traumatic stress disorder -PTSD (Zovkic et al., 2013).

### The Mouse Gambling Task: The Case of Decision-Making to Reveal Individual Strategies

The Mouse Gambling Task (MGT, Pittaras E. et al., 2016) is a decision-making task that we designed to study ambiguous and uncertain decision-making in mice. We were inspired from the human Iowa Gambling Task (IGT, Bechara et al., 1994). In this four-choice task, two choices give access to “advantageous” options in the long term, and the other two to “disadvantageous” options in the long term. The aim of the task is to earn as many food pellets as possible during the 100 trials (20 trials a day during 5 days).

During the habituation and the MGT, mice are food deprived and maintained at 85% of their free feeding weight.

**FIGURE 1:**
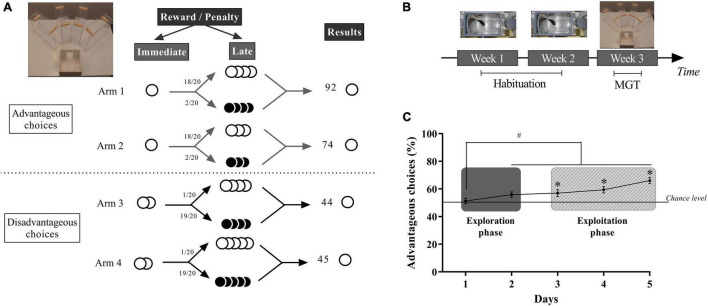
**(A)** Schematic representation of the MGT experimental design that takes place in a four-arm maze. In advantageous arms, mice find one pellet (small immediate reward) before a bottle cap containing three or four food pellets in 18 of the 20 trials and the same number of quinine pellets for the two remaining trials. In the disadvantageous arms, mice find two food pellets (large immediate reward) before a bottle cap containing four or five quinine pellets in 19 of the 20 trials and the same number of food pellets in the remaining trial. **(B)** Timing of the decision-making test: 2 weeks of operant chambers followed by 1 week of MGT. **(C)** Schematic representation of the mean of the percentage of advantageous choices of mice during 5 days of the MGT with illustrations of the exploration phase and the exploitation phase. *p < 0.05 from the chance level, #*p* < 0.05 differences between session 1 and the other sessions. Adapted from Pittaras E. et al. (2016) and Pittaras et al. (2018, 2019).

As described in more details previously (Pittaras et al., 2013), before starting the MGT mice were trained during 2 weeks in operant chambers. They learned to do a nose poke in an illuminated hole to obtain one food pellet during 30 min sessions once per day. This habituation phase was useful, but not mandatory, for the mice to get used to the experimenter, food deprivation, to eating food pellets as well as to perform an action to obtain food (Figure 1B).

The MGT was conducted as previously described (Pittaras E. et al., 2016). The task takes place in a maze with four transparent arms (20 cm long × 10 cm wide) containing an opaque start box (20 cm × 20 cm) and a choice area (Figure 1A). We used standard food pellets as a reward (dustless Precision Pellets, Grain-based, 20 mg, BioServ, Flemington, NJ, United States) and food pellets previously steeped in a 180-mM solution of quinine as a penalty. In “advantageous” arms mice systematically found 1 pellet (“small reward”) before a cup containing food pellets on 18 trials out of 20 and quinine pellets for two remaining trials (Figure 1A). In the “disadvantageous” arms mice found two food pellets (“large reward”) before a cup containing quinine pellets on 19 trials out of 20 and food pellets in the remaining trial (Figure 1A). Advantageous choices are at first less attractive than disadvantageous choices because of the small immediate reward (1 pellet vs. 2 pellets). Despite this apparent lower attractiveness, advantageous choices are advantageous in the long term because food pellets had higher probability of being found than quinine pellets. Conversely disadvantageous choices are less advantageous in the long term because animals had a higher probability of finding quinine pellets than food pellets. Therefore, mice had to favor the small immediate reward (advantageous choices) to obtain the highest amount of pellets as possible at the end of the day (Pittaras E. et al., 2016). Each trial began with the mouse placed in the starting box. The mouse can then freely choose one of the four arms and eat pellets at the end of it. We scored a choice when the mouse reached the middle of one arm. When the animal is done eating or loses interest in the food, it is put back in the starting box while the maze is cleaned. Therefore, mice learned quickly that if they chose one arm, they can’t go to explore another one during the same trial.

Each animal performed 20 trials per day: 10 trials in the morning (between 09:00 am and 01:00 pm) and 10 trials during the afternoon (between 02:00 pm and 06:00 pm). We scored the percentage of advantageous choices by day [(number of advantageous choices/number of total choices) × 100] and a rigidity score. The rigidity score is measured by looking at how many times the mouse chose the same arm. For example, a rigidity score of 25% means that the mouse chose an arm by chance and a rigidity score of 100% that the mouse always chose the same arm. Therefore, a rigidity score of 50% reflected that the mouse had chosen one arm twice as much as the others, and a rigidity score of 75% that animal had chosen one arm three times more often than the others (Pittaras E. et al., 2016).

In the MGT, we observed the development of mice’s preferences over time (Figure 1C): first they show an exploration phase in which they acquire information about each option, then this phase is followed by an exploitation phase in which mice use their knowledge about the putative value and risk associated with each option (de Visser et al., 2011a). These two phases can be determined by looking at the statistical difference between the chance level and the percentage of advantageous options as well as by looking at the behavioral strategies *vs*. random strategies of the mice. Results show that most of the mice eventually choose more long term advantageous options (Figure 1C).

### Statistical Analyses of the Data

**FIGURE 2:**
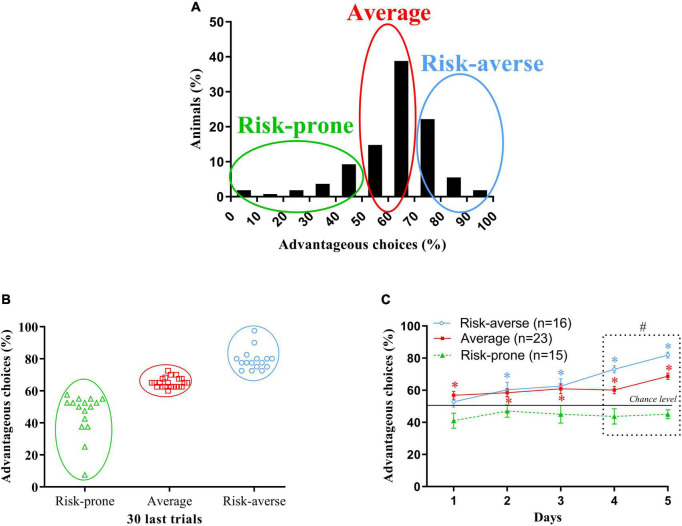
**(A)** Gaussian repartition of the percentage of animals depending on their preferences during the last 30 trials of the MGT. Risk-prone, average and risk-averse mice are circled. **(B)** Cluster proposed by the k-mean cluster analysis. Risk-prone, average and risk-averse mice are circled. **(C)** Evolution of the preferences of the risk-averse, average and risk-prone animals from sessions 1 through 5 in the MGT. *p < 0.05 from the chance level, #*p* < 0.05 differences from the three subgroups. Adapted from Pittaras E. et al. (2016).

Like in humans (Bechara et al., 2001), when we carried out the MGT, we observed an important variability regarding the preferences of mice at the end of the task: some animals, but not all of them, preferred advantageous options. Moreover, these inter-individual differences emerged only during the exploitation phase and, similar to what was observed in a healthy human population (Bechara et al., 2002), followed a Gaussian distribution (Figure 2A). Therefore, we asked if these differences come from the emergence of variable decision-making profiles in mice.

We next attempted to separate animals following the preference they show at the end of the MGT. Different approaches can be used to separate animals: mean ± Standard Deviation of the Mean (SEM), mean ± 2SEM, median ± SEM, median ± 2SEM, etc., but we decided to use the *k*-mean cluster analysis to rely on an unbiased statistical tool that guarantees an objective repartition of the animals (Timmerman et al., 2013). This statistical method places each animal in a subset that has the closest mean of preferences to its own preference value. One of the strengths of this method is that it repeats the statistical calculation several times to test all possible repartitions and proposes the best possible one. We used Statistica (version 12) to carry out the k-mean cluster analysis. The value we used to run the k-mean cluster analysis was the mean of preference calculated over the last 30 trials of the MGT (Figure 2B).

When we separate mice depending on their preferences at the end of the MGT, the k-mean cluster analysis distinguishes three distinct groups. We then looked at the evolution of the preferences of these three subgroups (Figure 2C):

- The majority of mice (44%, “average”) that preferred advantageous options without neglecting alternative—potentially riskier—choices,

- A small subgroup of mice (29%, “risk-averse”) that preferred long-term advantageous choices and progressively avoided exploring other options by developing rigid choice behavior, i.e., rarely changing options between two trials, and choosing arms associated with less quinine pellets,

- A small proportion of mice (27%, “risk-prone”) that continued to explore all available options throughout the experiment despite a low probability of getting a reward.

Therefore, the MGT combined with the k-mean cluster analysis allowed us to characterize three subgroups of animals showing distinct decision-making strategies.

### Behavioral, Neurobiological, and Neurochemical Bases

After establishing the three subgroups of mice showing different decision-making profiles, we wanted to determine if they also showed some behavioral, brain activity, or neurochemical differences (Pittaras E. et al., 2016).

#### Behavioral Traits

First, the three subgroups of mice did not differ regarding working memory, anxiety (dark/light), locomotor activity and exploration. Using the delay-reward task (Serreau et al., 2011), we also showed that risk-averse, average and risk-prone mice had the same ability to wait for a larger reward and they were all able to control their frustration. Therefore, the results of the risk-prone mice in the MGT were not due to inability to distinguish large from small rewards.

However, only risk-averse and average mice were sensitive to the reward by preferring drinking water with sucrose rather than regular water during the sucrose preference task (Ping et al., 2012). Moreover, like during the MGT, risk-prone mice also showed explorative and non-anxious behavior during the Elevated Plus Maze (Pellow and File, 1986) as risk-prone adolescent mice did compared to their adult counterparts, with no difference in risk assessment (MacrıÌ et al., 2001).

#### Brain Activity

Brain activities of the three subgroups were studied right after animals performed the MGT using cfos immunochemistry (Pittaras E. et al., 2016). No differences existed between the three subgroups regarding the activity of orbitofrontal cortex (OFC), amygdala, nucleus accumbens, basolateral amygdala, infralimbic, cingulate cortex, caudate putamen, insular cortex, hippocampus and motor cortex. However, risk-averse mice showed less activity in the prelimbic than risk-prone ones. Moreover, activity of the prelimbic cortex was correlated with the preferences of the mice as well as their rigid behavior of choice, called rigidity here. Risk-averse mice also showed less activity than the average group in the OFC and the nucleus accumbens.

#### Basal Level of Monoamines

Four months after completing all behavioral tasks, we measured the monoamine content in frozen dissected brain tissue using High Performance Liquid Chromatography (HPLC). Compared to risk-averse mice, risk-prone mice had higher levels of serotonin (5-HT), dopamine (DA), and noradrenaline (NA) in the hippocampus, and lower levels of 5-HT in the OFC (see Table 1).

**TABLE 1:** Statistical differences between risk-averse (blue, full arrow) and average mice and between risk-prone (green, dotted arrow) and average mice for basal levels of serotonin (5-HT), dopamine (DA) and noradrenaline (NA) in the orbitofrontal cortex (OFC), insular cortex, prelimbic, nucleus accumbens and hippocampus.

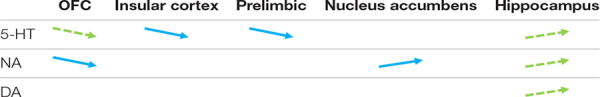

Additionally, risk-averse mice had lower levels of 5-HT in the prelimbic and insular cortex, as well as lower levels of NA in the OFC, and increased NA in the nucleus accumbens.

#### Conclusion

Risk-prone mice can be characterized by the fact that they showed riskier behavior in different behaviorals tasks and that they are less sensitive to reward. Risk-prone mice also showed a higher basal level of monoamines in the hippocampus, which could explain their exploratory behavior.

The risk-averse mice are characterized by a lower prefrontal activity, which agrees with the potential for more anxious behavior (Indovina et al., 2011) and less flexible choice (Granon and Floresco, 2009).

## Can We Shape or Reshape It?

The MGT allowed us to observe three separate groups of mice based on differences in their decision-making profiles. Moreover, these groups of mice are also characterized by different behavioral traits (more or less anxious or risk-prone behavior), brain activity (prefrontal hypoactivity for risk-averse mice) and basal monoamine levels (higher levels in the hippocampus for risk-prone mice).

We then asked how the environment can modulate these different decision-making profiles. Indeed, a stressful environment may lead to more anxious individuals and then to new decision-making profiles. On the contrary, an enriched environment could lower stress/reactivity level and therefore lead to a new repartition of animals in the three decision-making profiles.

To answer this question, we studied the effect of sleep debt, known to act on 5-HT levels (Bjorvatn et al., 2002), the effect of sweet or sweetener consumption and of enriched environment on the inter-individual differences during the MGT. We chose ecological ways to influence brain monoamine levels because our objective is to understand how environmental factors, classically present in human life, can influence decision-making processes.

### Effect of Sleep Deprivation

Some people are more vulnerable to sleep debt than others. These inter-individual differences in vulnerability to sleep loss are replicable and stable within individuals (Van Dongen et al., 2004; Rupp et al., 2012). These observations led to the hypothesis that vulnerability to sleep loss could be a trait or a phenotype (Rupp et al., 2012).

The Psychomotor Vigilance Task is widely used in humans (PVT, Van Dongen et al., 2004; Patanaik et al., 2014) to study sleep debt. It also has been shown that inter-individual differences in sustained attention are amplified following sleep debt (Doran et al., 2001). This suggests that sleep debt could be a trigger that emphasizes existing inter-individual differences during attention tasks (Chuah et al., 2014). One third of subjects had worse working memory scores after 30 h of sleep deprivation (Mu et al., 2005). This observation was linked to a difference in brain activity at baseline for individuals vulnerable or resistant to sleep deprivation: the global brain activity is more important for the resistant individuals at baseline and after sleep deprivation (Mu et al., 2005). Performance during a non-verbal memory task was also positively correlated with the level of brain activation (Bell-McGinty et al., 2004) as well as the Stroop test that showed that some people are resistant and others vulnerable to sleep debt regarding behavioral flexibility (Killgore et al., 2009). Moreover, performance in the go/no-go inhibition test was associated with increased activity of the right PFC and insula after 24 h of wake (Chuah et al., 2006). Regarding decision-making, only one study looked at the inter-individual variability after sleep deprivation (Caldwell et al., 2005). The performance of pilots in a flight simulator was measured after acute sleep deprivation. Some pilots were more resistant to sleep debt than others and this was correlated with global brain activity (Caldwell et al., 2005). Therefore, the level of prefrontal activation after sleep debt and at rest could be a marker of the vulnerability to sleep debt. Moreover, this prefrontal hypoactivity was also linked to elevated activity of the amygdala, an area that encodes emotional content of a situation.

Altogether, these data support the hypothesis of Doran et al. (2001) proposing that acute sleep debt (ASD) leads to a cognitive instability increasing inter-individual variability, most likely linked to hypoactivity in the prefrontal cortex at baseline, and under-inhibition of the amygdala. For the first time, we wanted to test this hypothesis in mice, which could be a useful tool to study the molecular basis of vulnerability to ASD.

Chronic Sleep Debt (CSD) also had a differential effect on attention in rats (Deurveilher et al., 2015). Some rats were resistant and others vulnerable to CSD. However, and contrary to what is observed in humans, attention deficits were only temporary in vulnerable rats. At the beginning of the CSD, animals showed a deterioration in their performance, but they improved with time to reach the level of control rats. Therefore, we wanted to compare the effects of ASD and CSD on decision-making in mice during the MGT as well as study the potential differential vulnerability to CSD in mice.

#### Acute Sleep Debt

As introduced above, decision-making is a set of processes highly sensitive to sleep debt in humans and some people are more sensitive to it than others. However, this vulnerability to sleep debt has been very little studied in animal models. Therefore, we used the MGT to study the inter-individual vulnerability to acute sleep debt (Pittaras et al., 2018). To do so, sleep was prevented during the MGT by using a transparent cylinder connected to a shaking platform that bounces randomly with variable frequency, intensity and duration (Viewpoint; Chauveau et al., 2014). The number and duration of the simulations were also randomly determined.

**FIGURE 3:**
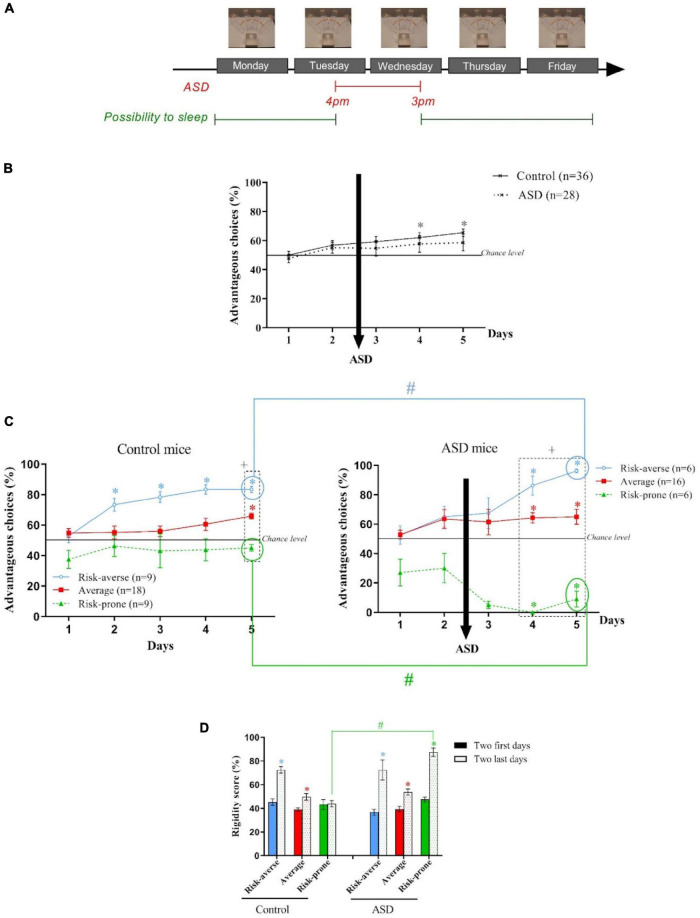
**(A)** Protocol of the Acute Sleep Debt (ASD) during the Mouse Gambling Task (MGT). **(B)** Evolution of the preferences of the animals as a group during the MGT for the control mice and the ASD mice. *p < 0.05 from chance level. **(C)** Evolution of the preferences of the risk-averse, average and risk-prone animals from days 1 through 5 in the MGT for the control and ASD mice. *p < 0.05 from chance level. + *p* < 0.05 differences from the three groups. #*p* < 0.05 differences between the three groups (Kruskal Wallis). **(D)** Rigidity score of the risk-averse, average and risk-prone animals at the beginning and the end of the MGT for the control and ASD mice. *p < 0.05 difference between the first two and last days. #*p* < 0.05 differences between control and ASD mice. Adapted from Pittaras et al., 2018.

ASD of mice during 23 h after the end of the MGT (day 5) had no effect on their decision-making performance and profile (Pittaras E. et al., 2016). Therefore, we decided to look at the effect of ASD before the establishment of decision-making profiles, i.e., before the exploitation phase (Figure 1C). As shown in Figure 3A, mice were subjected to ASD for 23 h.

As a group, mice were not able to establish preference for advantageous options after ASD and these observations were not linked to an increase of corticosterone concentration nor an increase of anxiety (Figure 3B). Regarding inter-individual differences, the k-mean cluster analysis led to the distinction of three subgroups: the risk-averse, average and risk-prone, but risk-averse animals significantly chose the more safe options, and the risk-prone mice chose the more risky options (Figure 3C). Rigidity scores of the risk-prone animals worsened after ASD (Figure 3D). Therefore, the existing inter-individual differences at baseline were amplified by ASD.

This study also showed that ASD disturbs brain neurochemistry with a decrease of 5-HT level in OFC and an increase of DA level in the dorsal striatum (Pittaras et al., 2018). Groman et al. (2013) had proposed that this neurochemical imbalance is associated with cognitive rigidity. Moreover, Faure et al. (2005) have shown that dopaminergic transmission in the dorsal striatum mediates habits formation. Therefore, the more rigid behavior we observed after ASD could be linked to the combination of a decreased 5-HT level in the OFC and an increased level of DA in the dorsal striatum.

In conclusion, ASD, when applied before the establishment of strategy (i.e., exploitation phase, Figure 1C), impairs decision-making in mice. When looking at the individual decision-making profiles, we discovered that 42% of mice are more vulnerable to ASD than others: risk-averse mice chose even more the safest options and risk-prone mice chose even more the riskiest options. This observation could be linked to the imbalance of 5-HT in the OFC and DA in the caudate putamen as well as the establishment of a rigid behavior.

#### Chronic Sleep Debt

**FIGURE 4:**
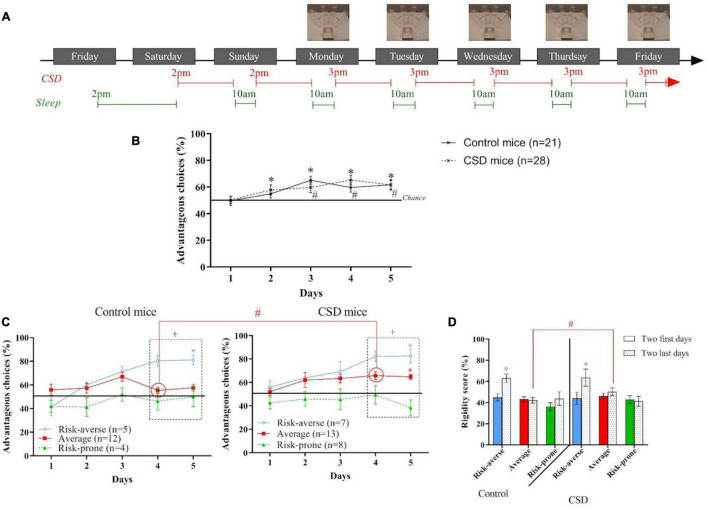
**(A)** Protocol of the Chronic Sleep Debt (CSD) during the Mouse Gambling Task (MGT). **(B)** Evolution of the preferences of animals as a group during the MGT for control mice and CSD mice. *and #*p* < 0.05 from chance level. **(C)** Evolution of preferences of risk-averse, average and risk-prone mice from days 1 through 5 in the MGT for control and CSD mice. *p < 0.05 from chance level. +, *p* < 0.05 differences from the three groups. #, *p* < 0.05 differences between the three groups (Kruskal Wallis). **(D)** Rigidity score of risk-averse, average and risk-prone mice at the beginning and the end of the MGT for control and CSD mice. *p < 0.05 difference between the first two and last two days. #*p* < 0.05 differences between control and CSD mice. Adapted from Pittaras et al., 2019.

We previously showed that ASD had a significant effect on decision-making and inter-individual differences during decision-making. Next, we investigated whether CSD during the MGT would also have deleterious effects. To accomplish this, we used the same paradigm as ASD (Chauveau et al., 2014), but the mice were allowed to sleep 4 h a day for 6 days instead of no sleep for 24 h (Figure 4A). Control mice were also placed in the apparatus for the same duration but the apparatus remained inactive.

As a group, CSD had no effect on mice; control and CSD mice were able to establish preference for advantageous options at the end of the task (Figure 4B). However, in CSD mice, there was a transient delay to reach significance from chance level, possibly due to the habituation of CSD timing (Figure 4B). A Gaussian distribution of mouse performance emerged during the MGT for control mice. Interestingly, we observed similar distribution, decision-making profiles and rigidity for CSD mice as well (Figure 4C).

In conclusion, we have shown that CSD does not interfere with decision-making strategies (Pittaras et al., 2019), which is likely due to mice adapting to CSD using a compensatory mechanism to maximize the beneficial effects of sleep during the hours when sleep is possible. Flexibility to habituate to a chronically stressful environment is also a possible contributing factor. Future work is needed to understand how this compensation is established, whether it is sustainable, and if other cognitive processes (e.g., attention, anxiety, etc.) can be altered.

### Influence of Sweet Consumption

As we have shown that manipulation of monoaminergic content can influence decision-making profiles, we chose to tailor the dopaminergic content of the reward system and prefrontal cortex by exposing mice to sweetened beverages. Indeed, sucrose intake has been shown to act on the reward system (Lenoir et al., 2007), modify brain monoamine levels and dopaminergic receptor expression, particularly in brain areas underlying decision-making processes (Smolders et al., 2007; Delaere et al., 2013; Ahmed et al., 2014; Naneix et al., 2016; Ren et al., 2020), and tailor monoamine gene expression in the prefrontal cortex, striatum and hippocampus (Harrel et al., 2015).

The consumption of sugar, particularly of sweet beverages, has become a major health issue (Lustig et al., 2012; Studdert et al., 2015). Indeed, consumption of sugar largely exceeds the recommendation of the World Health Organization (5% of the daily caloric needs, i.e., the equivalent of 6 teaspoons per day, 25g, OMS, 2018). However, although metabolic disorders associated with this consumption, such as obesity, type 2 diabetes, dyslipidemia, and cardiovascular diseases, are well described in humans (Pradhan, 2007; Lustig et al., 2012; OMS, 2018) and animal models (Leopoldo et al., 2010), much less is known about its effect on brain and cognitive functions. Some studies in humans highlight an impact of the hedonic value of sugar on psychiatric disorders such as bulimia (Goodman et al., 2018), schizophrenia or depression (Westover and Marangell, 2002; Peet, 2004; Pulgarón, 2013; Martin-Rodriguez et al., 2015; Knüppel et al., 2017) showing correlation -but not causality- between sweet consumption and the onset of disorders.

Therefore, we asked if sweet and sweetener consumption during 5 weeks could have an effect on the monoaminergic system that could lead to a modification of decision-making in mice and if some mice could be more vulnerable to this phenomenon.

#### Sugar Consumption

**FIGURE 5:**
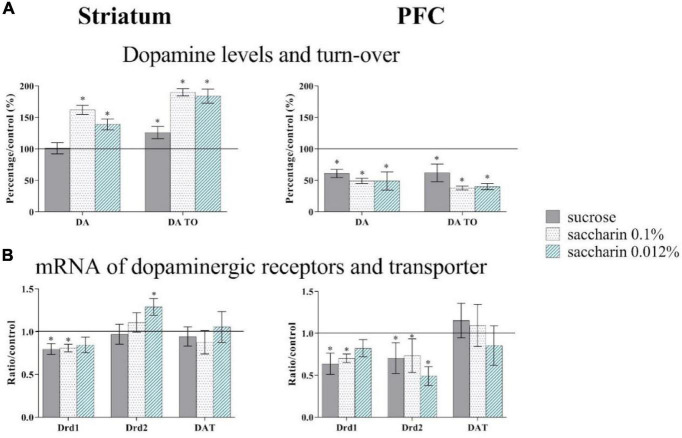
1% sucrose (gray bars), 0.1% saccharin (point bars) and 0.012% saccharin (striped bars) impact on dopamine levels (DA) and turn-over (TO) in HPLC quantifications **(A)** and on Drd1 (Dopamine receptor D1), Drd2 (Dopamine receptor D2) and DAT (Dopamine transporter) mARN (messenger ribonucleic acid) expression in RT qPCR **(B)** in the prefrontal cortex (PFC) and the Striatum. *p < 0.05. Adapted from Hamelin et al., 2021.

**FIGURE 6:**
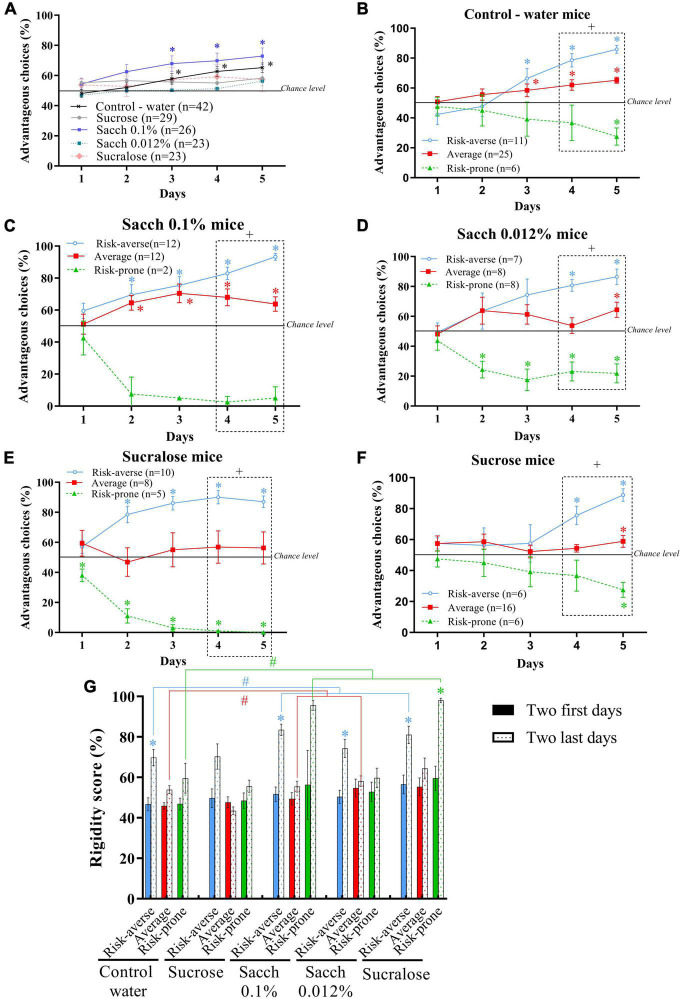
**(A)** Evolution of preferences of animals as a group during the MGT for mice drinking water, sucrose, Saccharin 0.1%, Saccharin 0.012% or sucralose. *p < 0.05 from chance level. **(B–F)** Evolution of preferences of subgroups of animals (risk-averse, average and risk-prone) from days 1 through 5 in the MGT for mice drinking water **(B)**, sucrose **(C)**, Saccharin 0.1% **(D)**, Saccharin 0.012% **(E)** or sucralose **(F)**. *p < 0.05 from chance level. + *p* < 0.05 differences between the three groups (Kruskal Wallis). **(G)** Rigidity scores of risk-averse, average and risk-prone mice at the beginning and the end of the MGT. *p < 0.05 difference between the first and last 2 days. #*p* < 0.05 difference between drinking groups. Adapted from Hamelin et al., 2021.

Our study has shown that continuous sugar consumption at a low dose (1% in home cage water, i.e., an increase of 25% from the daily amount) leads to behavioral, neurochemical and neuron activation disturbances (Hamelin et al., 2021; Figures 5, 6). Indeed, adult mice that consumed sugar for 5 weeks show decision-making alterations (Figure 6). Sugar consumption also leads to a decrease of approximately 40% in both the dopaminergic content and expression of the dopaminergic D2 receptor in the PFC, and a 21% reduction in the striatum (Figure 5). We also observed a modulation of neuronal activity in cfos immunochemistry following an appetitive task: sugar consumption reduces the neuronal activity in the PFC and in structures of the reward system (nucleus accumbens, OFC) and increases the activity of the basolateral nucleus of the amygdala (BLA, Hamelin et al., 2021).

#### Non-Metabolic Sweeteners Consumption

Over the last few decades, there has been a massive increase in the consumption of artificial sweet molecules without metabolic/caloric impact, such as saccharin, sucralose, and aspartame, to limit the deleterious effects of sugar consumption on metabolism. Currently, 25% of children and 40% of adults consume sugar substitute regularly (Sylvetsky et al., 2016; Pearlman et al., 2017). Discovered in 1879 by Constantin Faldberg (Arnold et al., 1983), saccharin was the first non-metabolic sweetener, and is commonly used in food as a substitute for sucrose (Shankar et al., 2013; Carocho et al., 2017). However, the non-metabolic impact of such substances is disputed and hitherto still studied (Fujita et al., 2009; Ford et al., 2011; Fowler, 2016; Olivier-Van Stichelen et al., 2019). The impact of sweetener consumption, and more particularly that of saccharin, on brain and cognitive functions have received little attention (Pepino, 2015). Some studies show that the consumption of non-metabolic sweeteners leads to eating behavior perturbations (Aoyama and Nagano, 2020; Awad et al., 2020) or alterations of learning and memory function (Erbaş et al., 2018).

In animal experiments, saccharin is classically used to control for caloric content of sucrose, as it mimics its sweet taste, with no metabolic or caloric consequences (Carroll et al., 2008 for review). Our recent study shows that in mice, continuous saccharin consumption at low doses (0.1% or 0.012%) leads to an increase of anterior insula activity and alterations of dopaminergic content, dopamine turn-over, and dopaminergic receptor expression in the prefrontal cortex and the dorsal striatum (Figure 5; Hamelin et al., 2021).

These neurobiological alterations following sugar or sweetener consumption may lead to different behavioral modulations depending on vulnerability in mice.

#### Individual Vulnerability to Sweet and Sweetener Consumption

When looking at individual choice performance of animals consuming sucrose or sweeteners, we observed that 1% sugar, or 0.012% saccharin or sucralose consumption led to no preference for advantageous choices in the MGT (Figure 6A), while mice consuming 0.1% saccharin improve their performances by displaying an increase in preference for advantageous options as well as a decrease in latency for this preference (Figure 6A) compared to control mice consuming water.

As previously observed in healthy and ASD mice, the study of inter-individual variability leads to a distribution of three subgroups using the k-mean cluster analysis: risk-averse, average and risk-prone mice following sweet or sweetener consumption (Figures 6B-F).

With the control -water consuming- group, we replicated the previously described results (Pittaras et al., 2013, 2018, 2019; Pittaras E. et al., 2016; Figure 6B) and showed that the sweet or sweetener consumption led to modifications of the different strategies of decision-making (Figures 6C-F). Indeed, saccharin and sucralose consumption accelerates the development of the different strategies, with mice exploring only one day before choosing their strategies (Figures 6C,D). We observed the same evolution with sucralose consumption (Figure 6E), showing that this pattern of behavior is not related to the molecule consumed but to the sweet taste it generates. However, the proportion of mice in each sub-group varies between each treatment. We observed that the majority of mice that consumed the more concentrated dose of saccharin (0.1%) avoided risk taking: only two mice are in the risk-prone subgroup whereas the others prefer the risk-averse choices (Figure 6C). For the less concentrated doses of saccharin (0.012%) or sucralose treatments, a higher proportion of mice preferred the riskiest choices. In the 0.012% saccharin treatment, mice were equally distributed in each subgroup (Figure 6D), and in the sucralose treatment, the performance of mice in the average group didn’t differ from chance level (Figure 6E), revealing increased risk-taking in this group.

Sugar consumption led to delayed strategies (Figure 6F) and more risk-prone behavior, with a large proportion of mice preferring the riskiest choices: mice belonging to the average subgroup didn’t show preference for advantageous choices and remained at chance level for 4 sessions.

Lastly, we have studied the rigidity of choice following sweet and sweetener consumption as previously described (Figure 6G; Pittaras et al., 2013, 2018, 2019; Pittaras E. et al., 2016). We show that sweetener consumption leads to an increase in mice’s rigidity, whether they exhibit risk-averse or risk-prone decisions.

In conclusion, we have demonstrated that sweet or sweetener consumption leads to modifications in decision-making strategies without drastically remodeling the pattern previously obtained in healthy mice. Indeed, three subgroups were observed. These results show that some mice were more vulnerable to the sweet or sweetener consumption than others, and developed more risk-averse and rigid choices, or took more risks. So far, we are searching for the mechanisms underlying the individual vulnerability. We were able to show that it cannot be explained by metabolic alterations. Indeed, we measured various biological markers (insulinemia, glucose tolerance, insulinemia, sweet taste receptor expression in the brain…) that were not different between groups of consumption. However, it cannot be assessed with the current methods by which mechanisms animals eventually become risk-averse or risk-prone after sucrose or sweetener consumption, nor have we yet studied the reversibility of these effects. This is actually the issue of current research in our laboratory.

### The Effect of Enriched Environment

We have seen that sleep debt and sweet/sweetener consumption can shape decision-making strategies in healthy adult animals. We then asked whether being exposed to a stimulating environment could also shape decision-making. The hypothesis behind this manipulation relies on the fact that novelty exploration is promoted by exposure to novel and enriched environments -as compared to impoverished ones in laboratory animals. As we and others have shown, the exploratory phase is of major importance for the discovery of options in decision-making tasks (Humphries et al., 2012), we hypothesized that manipulating this phase would influence decision-making performance.

For rodents, an enriched environment was described by Rosenzweig et al. (1978) as “a combination of inanimate and social stimulation” (Rosenzweig et al., 1978; Kempermann, 2019) in housing conditions, which tend to facilitate enhanced sensory, cognitive, motor and social stimulation relative to standard housing conditions (van Praag et al., 2000). In the last few decades, the number of studies on enriched environments has largely increased. Thus, studies show that enriched environments in rats or mice improve cognitive function and neuronal plasticity (van Praag et al., 2000; Bardi et al., 2016; Kempermann, 2019; Crawford et al., 2020). However, inter-individual variability was less scrutinized in the context of an enriched environment. Freund et al. (2013) show that 3 months of living in an enriched environment massively increased the individual differences in explorative behavior among genetically identical mice. Körholz et al. (2018) also show that enriched environments increase the inter-individual variability among a population of C57Bl/6J mice.

Here, we report novel data obtained in mice that were placed in enriched cages for 16 h per day, every day during 3 weeks. In enrichment cages, male C57BL/6J mice (*n* = 16), aged 10 to 12 weeks, found novel items and stimuli placed to favor locomotor activity (running wheel), olfactory stimulations (different spices in tea balls), auditory sense (classical music played during the first 3 h), and a wide range of toys that were changed every day (several different LEGO toys, nesting material, plastic tubes, plastic houses, etc.). Except the EE, the habituation and the MGT were performed exactly the same way as for the ASD, CSD and the sugar/sweetener consumption (see above). We compared MGT performance in mice placed in this device (EE mice, *n* = 16) and mice maintained in standard rearing facilities (Control mice, *n* = 24).

**FIGURE 7:**
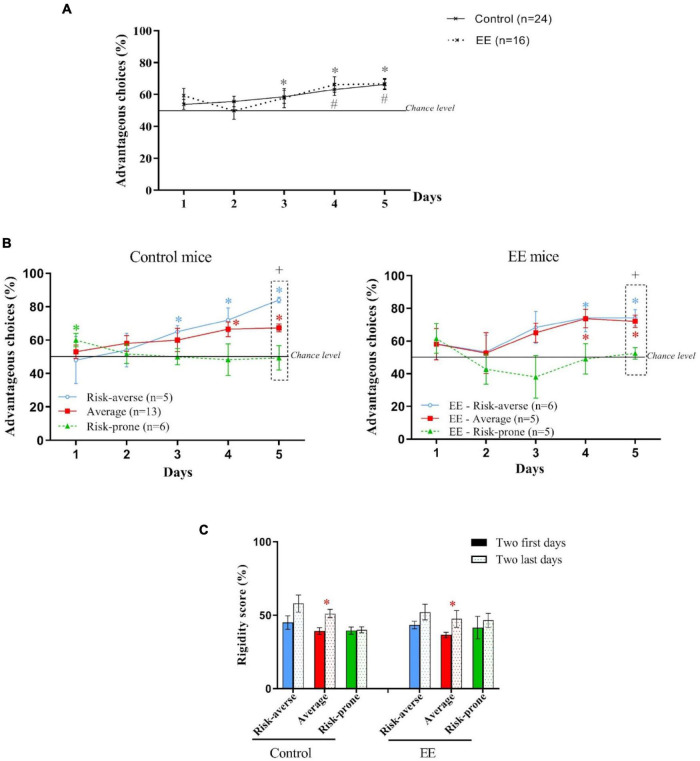
**(A)** Evolution of preferences of animals as a group during the MGT for Control and EE mice. *and # *p* < 0.05 from chance level. **(B)** Evolution of preferences of risk-averse, average and risk-prone mice from days 1 through 5 in the MGT for Control and EE mice. *p < 0.05 from chance level. +, *p* < 0.05 differences from the three groups (Kruskal Wallis). **(C)** Rigidity scores of subgroups at the beginning and the end of the MGT for the control and EE mice. *p < 0.05 difference between the first and last 2 days.

As shown in Figure 7A, Control and EE mice progressively chose the long term advantageous options across days during the MGT. However, EE mice started to choose advantageous options earlier than the Control mice. As observed before, the *k*-mean cluster analysis leads to the distinction of three subgroups of Control mice: the risk-averse, average and risk-prone (Figure 7B). However, EE mice only gave rise to two statistically distinguishable subgroups: the risk-prone and the average (Figure 7C). Indeed, risk-averse mice behave like average mice and couldn’t be dissociated statistically. Also, only average mice -but not risk-averse mice- showed increased rigidity after EE (Figure 7D). Therefore, an EE environment led to a decreased proportion of animals showing a rigid, non-flexible behavior during the MGT.

In conclusion, these unpublished results showed that the EE did not affect decision-making strategies as a group but led to different profiles of decision-making, mostly due to a major decrease of rigidity.

## Consequences on Individual Vulnerability to Pathological States

### Addiction

Within the global healthy population, only some individuals will develop drug addiction. Vulnerability to develop addiction depends on behavioral traits of the individual, the drugs and the environment of the individual (Belin and Deroche-Gamonet, 2012; Homberg et al., 2014). For example, the positive or negative emotionality as well as the constraint of an individual have been linked to dopamine and serotonin system and substance use disorders (Belcher et al., 2014). A large number of very interesting reviews are published on vulnerability *vs.* resilience to drug addiction (e.g., Swendsen and Le Moal, 2011; Egervari et al., 2018; Miela et al., 2018) and anxiety, sensation-seeking and impulsivity are proposed to be strongly correlated to excessive drug consumption (Belin et al., 2016). For example, when the sensation-seeking behavioral trait is detected in an adolescent, it could predict adult alcohol and tobacco consumption (Crawford et al., 2003; Sargent et al., 2010). However, behavioral traits evolve during a lifetime and also during drug consumption.

Drug addiction can be measured by looking at three criteria: 1- inability to refrain from drug seeking, 2 - high motivation for the drug, and 3 - compulsive drug use despite negative consequences (Belin et al., 2011). Sensation-seeking was modeled in rodents by looking at the research of novelty in a new environment (Dellu et al., 1996). Rats that showed a high locomotor response to the exposure of a new environment also had a higher tendency to self-administer drugs (Piazza et al., 1989, 2000). Moreover, rats that showed a phenotype in which they preferred a new environment compared to a known one, were also those that compulsively used drugs (Belin et al., 2011). In addition to sensation-seeking, anxiety and impulsivity have also been shown to be linked to addiction (Belin et al., 2016). Indeed, anxious individuals self-regulate their distress by using drugs (Lejuez et al., 2008). This emotional self-regulation could be linked to the initial drug use, the inability to refrain from drug seeking as well as the continuity of drug taking (Belin et al., 2016). Anxiety can be measured in animals by using the Elevated Plus Maze (EPM; Pellow et al., 1985). This paradigm can also be used as a measure of risk taking (Rodgers and Johnson, 1995). The more rodents showed anxious behavior during this task, the more they will self-administer themselves cocaine, and the more they will have a preference for alcohol (Belin et al., 2016). Finally, impulsivity is also a behavioral trait linked both to the development of addiction and to the tendency to develop compulsive drug consumption (Belin et al., 2008). Therefore, rats that are more impulsive during the delay-reward test (cognitive impulsivity) or the 5-choice serial reaction time task -CSRTT motor impulsivity- will develop drug self- administration behavior faster (Belin et al., 2016).

At the molecular level, impulsive animals, without any consumption of drugs, have less dopamine receptors (D2/D3) in the ventral, but not in the dorsal striatum (Dalley et al., 2007). This result shows that availability of dopaminergic receptors could be a molecular marker of the vulnerability to addiction. The brain network involved in vulnerability to drug addiction has been proposed to recruit several brain areas including the PFC, nucleus accumbens, caudate putamen, etc. (Domingo-Rodriguez et al., 2020; Ersche et al., 2020), but more research is still necessary.

Thanks to the MGT, we showed that in a healthy mice population, some mice maintained exploration of available options even if this strategy was less rewarding: the risk-prone mice. These mice were also characterized by riskier behavior during the EPM and less sensitivity to reward during the sucrose preference task, but did not show any difference regarding their memory, impulsivity, locomotion, and ability to distinguish between a large and a small reward (Pittaras E. et al., 2016). Therefore, the main behavioral characteristic of these mice seem to be that they are more prone to exploration and sensation-seeking.

At a molecular level, these mice showed a high basal rate of monoamines in the hippocampus that might prevent them from establishing an appropriate action-outcome relationship as food reinforcement is associated with a decrease in DA and 5-HT in the hippocampus and prefrontal cortex (González-Burgos and Feria-Velasco, 2008). DA and 5-HT in the hippocampus are also involved in learning and memory (González-Burgos and Feria-Velasco, 2008). Therefore, risk-prone mice may be more prone to explore and learn spatial cues, hence to rely on external information rather than internal ones by maintaining exploration phase.

Sensation- seeking, risk-taking and high reactivity to novelty predicts a propensity to initiate cocaine self-administration (Belin et al., 2008, 2011). In addition, level of 5-HT in the OFC plays a key role during top-down control of decision-making (van den Bos et al., 2013). We showed that exposing continuously healthy mice to sucrose or sweetener changes total DA content in the PFC and striatum, dopaminergic receptor expression and that risk-proneness can be manipulated by sucrose beverage exposure (Hamelin et al., 2021). This pattern of behavior is also observed in animals subjected to ASD: sleep doesn’t change the proportion of risk-prone animals, but rather worsens their tendency for risk-taking (Pittaras et al., 2018) and imbalances the DA and 5-HT system in the OFC and the caudate putamen. Altogether, these results showed that risk-prone mice share behavioral and neurochemical characteristics that are also found in individuals vulnerable to drug addiction. Therefore, we propose that risk-prone mice, identified in the MGT, could be good models for vulnerability of addiction.

### Anxiety/Depression

Fear or stress are normal physiological adaptive behaviors when an individual is in a dangerous environment. However, experiencing these sensations for a long time or in a non-dangerous environment could lead to anxiety disorders or depression. In everyday life, people are generally subjected to socio-professional stress. However, not all of them develop anxiety disorders or depression. Therefore, some people are more sensitive to developing these pathologies than others and, like addiction, it is linked to a combination of individual behavioral traits and the living environment.

In humans, one of the behaviors that is associated with the development of depression, the severity and the duration of anxiety/depression is rumination (Nolen-Hoeksema, 1991; Nolen-Hoeksema et al., 1993; Just and Alloy, 1997). Individuals who have frequent negative thoughts are more vulnerable to depression according to the cognitive vulnerability hypothesis (Alloy et al., 2006). Indeed, cognitive vulnerability is observed when an individual negatively reacts to stressful events in life by thinking that it is impossible for them to improve their situation because of their ineptitude. Therefore, these people will develop depression more easily (Alloy et al., 2006). Another behavioral trait that could be linked to anxiety and depression is anhedonia, which is an incapacity to feel positive emotion. In animal models, anhedonia can be tested by using the sucrose preference task that is based on the principle that animals will usually prefer sweet solution over water. The forced-swim test is also a way to study depression-like-behavior in rodents (Porsolt et al., 1978). During this task, the animal is placed in a beaker full of water and the duration the animal swims to avoid sinking is measured.

Animals that are more anxious will develop depression faster, when submitted to stress, than the ones that are less anxious (Castro et al., 2012). This behavioral result was linked to higher corticosterone levels after a stressful event (Castro et al., 2012). At the molecular level, the ventro-medial PFC inhibits fear expression by modulating the activity of the amygdala (Indovina et al., 2011). In other words, the more an animal is anxious the more its prefrontal activity will decrease in a fear context, and this will lead to an over activation of the amygdala (Indovina et al., 2011). Therefore, the basal activity of the PFC of an individual could explain how sensitive he could be in front of a stressful situation.

In our experiments, we showed that risk-averse mice strongly preferred advantageous options during the MGT (Pittaras E. et al., 2016). The magnitude of this preference and the rapidity for establishing it in a particularly non-flexible way was even reinforced after sweetener exposure (Hamelin et al., 2021). However, risk-averse mice did not systematically choose the arm associated with the larger reward and did not earn more pellets than average mice: they reduced exploration of other options, and exhibited rigid behavior. Since risk-averse mice recognize the value of a reward in the sucrose preference test as well as in the delay reward task (Pittaras E. et al., 2016), their choices in the MGT are likely guided by penalty avoidance, to the detriment of exploration and flexibility. This hypothesis is reinforced by our data obtained after sweetener exposure (Hamelin et al., 2021) as this consumption triggers a massive and early increase in risk-averse choices, also increased insula-based network activity and perturbed DA levels in the prefrontal-striatal network. The monoamine pattern of risk-averse mice is congruent with results obtained in monkeys showing inflexible behaviors associated with regional imbalance of DA and 5-HT (Groman et al., 2013).

Anxious subjects performing a risk-prone decision-making task exhibited hypoactivation of the PFC in loss condition (Galván and Peris, 2014). Moreover, lesioning the OFC or PrL leads to unadapted decision-making (Granon et al., 1994; Rivalan et al., 2011; Granon and Changeux, 2012), and the exploration phase in the MGT requires the activation of the limbic loop, while the exploitation phase requires the activation of the cognitive loop at the cost of the limbic loop (de Visser et al., 2011b; Koot et al., 2013). Notably, risk-averse mice exhibited reduced activation of the cognitive loop, specifically the PrL area, compared to other subgroups that are necessary for flexible behaviors (Boulougouris et al., 2007; Floresco et al., 2009; Granon and Floresco, 2009; Young and Shapiro, 2009; Mihindou et al., 2013). The activity of the PrL of the mice was correlated with their performance during the last 30 trials and to their rigidity scores, thus illustrating the fact that a low PrL activity is expected to be a marker of rigid behavior (Granon and Changeux, 2012). Hypo-activation in risk-averse mice of brain regions involved in the integration of both limbic and cognitive information could explain their elevated rigidity score at the end of the task.

These results show that individual patterns of choice can be easily influenced by environmental factors. In a healthy -and congenic- mouse population, some mice favor risk-averse strategies to avoid possible negative consequences. Although our risk-averse mice did not show a general higher level of anxiety in our experimental conditions, their propensity to prefer conservative and rigid choices are possible traits that increase their vulnerability to anxiety. Moreover, an enriched environment (EE) leads to a decrease in the percentage of risk-averse mice. These results are in line with the evidence that EE decreases symptoms of depression, anhedonia and anxiety caused by early maternal separation (Huang et al., 2021). However, the elevated vulnerability of risk-averse mice to depression and anxiety remains to be fully investigated.

## Concluding Remarks

We reviewed here data showing inter-individual variations in the Mouse Gambling Task (MGT), a task that allows the progressive development of individual choice strategies in an environment with an uncertainty component, which matches most real life context (van den Bos et al., 2013). Using large groups of animals is the only sound option to reveal clustering of animals, based on their decision-making performance with unbiased statistical methods. We repeated these data multiple times in healthy animals, demonstrating their robustness and the need for studying a large number of individuals. This cluster of animals showed behavioral and neurochemical specific characteristics.

**FIGURE 8:**
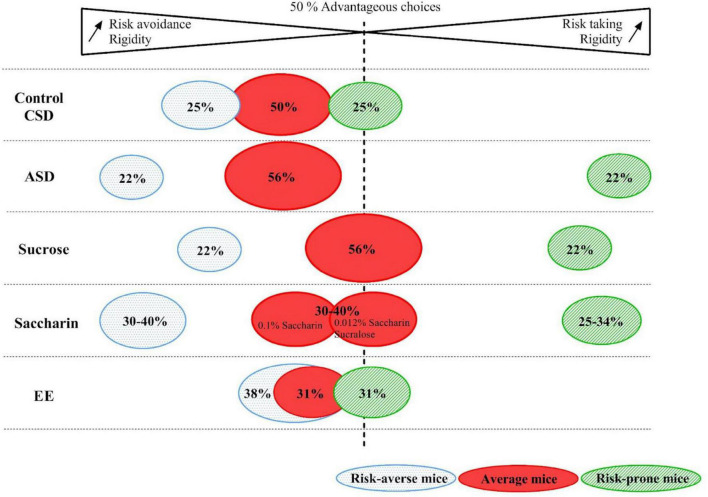
Representation of the percentage of risk-averse, average and risk-prone mice in the control, Chronic Sleep Debt (CSD), Acute Sleep Debt (ASD), sucrose or saccharin consumption and Enriched Environment (EE) conditions. Gradients of rigidity as well as risk taking or risk avoidance are represented by the arrows.

Individual profiles established in this task show that risk-proneness or risk-aversion is a critical behavioral marker of decision-making (see Figure 8 for a schematic representation of MGT individual profiles). The relation between risk-taking (including sensation or novelty seeking), anxiety, and personality traits has been studied by several authors who provided very interesting data showing that the brain monoamines underlie behavioral mechanisms related to personality traits (for review see Zuckerman and Kuhlman, 2000). To that regard, the MGT is promising for translational studies, with excellent construct validities. In addition, it provides an easy way to determine individual risk-taking profiles to study associated behavioral, neural, physiological, and possibly, epigenetic markers. One of the behavioral aspects that we didn’t include in our current review -as it would require a full paper- is the relationship with social environment/social traits. This also would remain to be included in the search for environmental factors that participate in the building of individual cognitive strategies. For example, risk-taking behaviors that may lead to negative consequences, have also been shown to be important for the stability of social structure, proving dynamic flows between individuals and incentives (Sapolsky, 2005). In addition, risk-taking behaviors are important components in the allocation of resources with social hierarchies. The increased risk-taking/novelty seeking behaviors has been shown to be higher in adolescents than in adults and to be strongly modulated by the development of the ascending monoaminergic system (for review see Laviola et al., 2003). As we showed a relationship between the level of cortical monoamines and risk-related gambling behaviors, we can expect social individual features to impinge on the development of individual decision strategies.

Studying inter individual decision making can also allow us to study how environmental factors can help shaping them and how individual ability could be improved or impaired. The MGT is a very interesting tool to study these questions. Indeed, during the MGT, decision-making profiles are responding differently to stressful, dietary modification or a good environment. CSD had no effect on decision-making but ASD led to an exacerbation of extreme profile without changing the Gaussian repartition of the mice. The sucrose consumption also did not change the repartition of the mice but slightly increased the rigidity of the extreme profile of the mice and, more importantly, altered the decision-making of average mice. Conversely, the saccharin consumption radically changes the repartition of the mice, as well as their rigidity, by increasing the percentage of risk prone and averse mice groups. Regarding the effect of a pleasant/stimulant environment (EE), the rigidity of mice decreased as well as the percentage of extreme profile risk-averse mice.

Therefore, an acute short stress seems to be enough to drive mice, already showing a moderate extreme behavior, to maladapted decision-making strategies and a rich environment is enough to decrease/suppress the percentage of mice, showing a rigid/risk-averse behavior. On the contrary, subjecting mice to sweetener drinking solutions even increases the percentage of mice presenting an extreme maladaptive behavior. However, subjecting mice to sweet drinking solutions affected only the average mice and CSD had no effects on decision-making. These observations point out how the environment has an important effect on decision-making and how its quality could lead to extreme “pathological” decision-making profiles. Indeed, after ASD or sweeteners consumption mice showing behavioral and neurochemical characteristics also found in individuals vulnerable to addiction or anxiety/depression emerge and EE decreased the propensity to see the emergence of these “pathological” profiles. One of the questions we may ask concerns the dynamic of the establishment, the stability and the translation to other brain processes, of individual cognitive strategies. Most studies we reviewed have not addressed this issue and this would be the next challenge. This question is related to the next one, which concerns the use of individual traits to predict vulnerability/resilience to pathological conditions, as illustrated above with the examples of addiction and anxiety-related disorders. This also remains to be fully investigated.

Studying individual behavioral and biological profiles doesn’t discard, in our opinion, group studies. Indeed, global group comparison may provide a general effect of a manipulation (e.g., effect of a constitutive mutation, Pittaras E. C. et al., 2016), before giving access to individual vulnerability traits (e.g., after sleep deprivation, Pittaras et al., 2018), as some effects may affect the whole population but may also be tampered by individual physiology or history and highlight behavioral and brain plasticity.

The reviewed data, and particularly those obtained from environmental manipulations (Figure 8), suggest that being exposed to an adequate environment (stimulating, offering sufficient sleep opportunities and devoid of exaggerated sweet stimulants carrying no nutritional value, for example) can promote the establishment of a mental/cognitive reserve (see Pettigrew and Soldan, 2019; Stern et al., 2020 for reviews), supported by balanced brain monoamine levels. This area of research is of particular interest for studying healthy aging (Ferreira et al., 2017; Vallet et al., 2020), which is shown to be associated with some cerebral markers such as resting-state brain activity (Bastin et al., 2012), and prefrontal network and connectivity development during adolescence, a period of life in which the cognitive reserve may be most vulnerable to environmental challenges (Blakemore and Robbins, 2012; Larsen and Luna, 2018). We expect this review to open novel ways of looking at behavioral data, and to consider individual brain-behavior relationships as the foundation of brain plasticity, both in real life and in the laboratory.

### Take Home Message

The Mouse Gambling Task revealed about 30% of healthy mice displaying risk-averse choices while about 20-25% of mice make risk-prone choices. These strategies are accompanied by different brain network mobilization and individual levels of regional -prefrontal and striatal- monoamines. We also illustrate three ecological ways that influence drastically cognitive strategies in healthy adult mice:

• acute sleep deprivation, that behaviorally exacerbes already exciting extreme decision-making profiles,
• artificial sweetener exposure that increases the proportion of mice showing extreme decision-making profiles,
• exposure to a stimulating environment that decreases the proportion of mice showing extreme decision-making behavior.

Conduction of correlations across multiple biological markers obtained in large cohorts (multiple behavioral data, neurochemical, brain activity, metabolic…) is a valuable approach that may open new avenues for the identification of vulnerability traits to adverse events, before the emergence of mental pathologies.

## Author Contributions

EP, HH, and SG wrote the manuscript. All authors contributed to the article and approved the submitted version.

## Conflict of Interest

The authors declare that the research was conducted in the absence of any commercial or financial relationships that could be construed as a potential conflict of interest.

## Publisher’s Note

All claims expressed in this article are solely those of the authors and do not necessarily represent those of their affiliated organizations, or those of the publisher, the editors and the reviewers. Any product that may be evaluated in this article, or claim that may be made by its manufacturer, is not guaranteed or endorsed by the publisher.
